# Complete genomes of hybrid *Escherichia coli* isolates containing virulence factors from enterotoxigenic and enteropathogenic pathovars

**DOI:** 10.1128/mra.00959-25

**Published:** 2025-11-10

**Authors:** Bernadette A. Hritzo, Jane M. Michalski, Michael J. Sikorski, David A. Rasko

**Affiliations:** 1 Institute for Genome Sciences, University of Maryland School of Medicine 12264, Baltimore, Maryland, USA; 2 Department of Microbiology and Immunology, University of Maryland School of Medicine 12264, Baltimore, Maryland, USA; 3 Department of Microbial Pathogenesis, University of Maryland School of Dentistry 89015, Baltimore, Maryland, USA; 4 Center for Vaccine Development and Global Health, University of Maryland School of Medicine 12265, Baltimore, Maryland, USA; 5 Center for Pathogen Research, University of Maryland School of Medicine 12264, Baltimore, Maryland, USA; Nanchang University, Nanchang, Jiangxi, China

**Keywords:** *Escherichia coli*, hybrid, pathogen

## Abstract

This announcement describes the genomes of three hybrid *Escherichia coli* isolates containing virulence factors from enteropathogenic and enterotoxigenic *E. coli*. The recognition and characterization of these hybrid pathogens is essential in understanding the development of these novel organisms as an emerging threat among enteric pathogens.

## ANNOUNCEMENT

Pathogenic *Escherichia coli* are often categorized into six generally accepted pathogenic variants (1, 2), or pathovars, each characterized by the presence or absence of virulence-related genes. Recent studies (3–7) and outbreaks (4, 8, 9) demonstrate that *E. coli* isolates containing virulence factors from multiple pathovars, termed hybrid *E. coli,* have infected humans. This announcement describes the genomes of two hybrid *E. coli* isolates obtained as part of the Global Enteric Multicenter Study (GEMS) (10) and an additional isolate from Bangladesh (11). The isolates contain the heat-labile toxin from enterotoxigenic *E. coli* (1, 2) and the type 3 secretion system from enteropathogenic *E. coli* (1, 2). Isolates produce each of the pathotype virulence factors, as demonstrated by our previous functional characterization (12).

The *E. coli* isolates were initially isolated as described by Panchalingam et al. (13). Briefly, feces or a rectal swab were transported in cold containers and inoculated into Cary-Blair transport media within 6 hours; within 18 hours of collection, samples were inoculated into selective growth media for multiple pathogens. For *E. coli,* several lactose-fermenting bacterial colonies were selected from McConkey agar plates incubated for 48 hours at 37°C. Colonies were sub-cultured in MIO medium, and indole-negative cultures were further assessed by Methyl Red, Voges-Proskauer, and Citrate biochemical tests. Suspected *E. coli* isolates (methyl-red positive; Voges Proskauer- and citrate-negative) were categorized into the pathogenic types by multiplex PCR (14). Verified *E. coli* isolates were frozen in 20% glycerol and maintained at −80°C. A frozen culture was provided to, and stored at, the Center for Vaccine Development at the University of Maryland School of Medicine.

Isolates were resurrected from frozen culture on Lysogeny agar overnight at 37°C. Genomic DNA was extracted using Qiagen’s DNeasy Blood & Tissue kit, and paired-end sequencing libraries were generated with unsheared genomic DNA. Nanopore libraries were prepared using genomic DNA that was not sheared or size selected with Oxford Nanopore’s “Genomic DNA by Ligation” kit and protocol (SQK-NBD114.24) (15). All samples were run on Nanopore R9 flow cells on a MinION Mk1B, and basecalling was performed using Guppy (v4.2.2) (16). Illumina libraries were generated using the Kapa library kit (Roche/Illumina) and sequenced with 2 × 150 bp chemistry on the Illumina HiSeq 4000 platform. Quality control and adapter trimming were performed with bcl2fastq (v2.20.0.445) (17) and porechop (v0.2.3_seqan2.1.1) (18) for Illumina and ONT sequencing, respectively. Hybrid assembly using both Illumina and ONT reads was conducted with Unicycler (v0.4.8) (19), including circularization. The final assemblies were not rotated to a specific base, and statistics were recorded with QUAST (v5.0.2) (20). The assemblies were then annotated with the NCBI prokaryotic genome annotation pipeline (v6.10) (21). All software was run with default values unless otherwise specified. Table 1 contains the total number of reads generated for each isolate and sequencing technology, relevant sequencing statistics, and GenBank accession numbers of each assembly.

**TABLE 1:** Isolate, sequencing, and assembly metrics

Samples			Sequencing and assembly metrics											GenBank accessions		
Sample	Country	Case/control	Illumina read pairs	Illumina bases	Nanopore trimmed reads	Nanopore trimmed bases	N50 Nanopore	Coverage	No. of molecules	Total genome length (bp)	GC (%)	Molecule name	Size (bp)	BioProject	Assembly	SRA (Illumina, ONT)
602687	Bangladesh (GEMS)	Case	3,661,295	1,131,117,901	1,022,938	1,340,426,141	1,346.9	477.3	5	5,178,254	50.66	E602687	5,023,812	PRJNA1254407	CP189657	SRR33281816
												pE602687_136	136,111		CP189658	SRR33281813
												pE602687_12	12,678		CP189659	
												pE602687_3	2,975		CP189660	
												pE602687_2	2,678		CP189661	
102651	Gambia (GEMS)	Case	3,209,872	988,252,708	413,151	1,153,268,001	2,821.9	413.5	8	5,179,396	50.78	E102651	4,889,307	PRJNA1254407	CP189662	SRR33281817
												pE102651_87	87,234		CP189663	SRR33281814
												pE102651_75	74,718		CP189664	
												pE102651_74	73,814		CP189665	
												pE102651_41	41,391		CP189666	
												pE102651_5	5,539		CP189667	
												pE102651_4	4,087		CP189668	
												pE102651_3	3,306		CP189669	
2854350	Bangladesh	Case	3,653,491	1,124,926,571	2,582,536	2,492,782,242	1,008.8	702.8	5	5,147,275	50.74	E2854350	4,940,102		CP189652	SRR33281815
												pE2854350_145	145,467		CP189653	SRR33281812
												pE2854350_52	52,382		CP189654	
												pE2854350_5	5,126		CP189655	
												pE2854350_4	4,198		CP189656	

The identification and characterization of these hybrid isolates will allow a greater understanding of these emerging pathogens, which can inform effective therapeutics and diagnostics.

## Data Availability

All data can be accessed in BioProject PRJNA1254407, and the associated SRA and genome assembly accession numbers are listed in Table 1.
